# Available energy content, nutrients digestibility of chili meal and effects on performance of growing pigs

**DOI:** 10.1016/j.anifeedsci.2017.05.001

**Published:** 2017-07

**Authors:** Y.F. Fan, Y.Y. Yang, P. Yang, T. Xia, Y.X. Ma

**Affiliations:** State Key Laboratory of Animal Nutrition, Ministry of Agriculture Feed Industry Centre, China Agricultural University, Beijing 100193, China

**Keywords:** ADFI, average daily feed intake, ADG, average daily gain, ALB, albumin, ALP, alkaline phosphatase, ALT, glutamic-pyruvic transaminase, AST, glutamic oxalacetic transaminase, CM, chili mea, CREA, creatinine, F:G, feed gain ratio, CLB, globulin, GLU, glucose, HDL-C, high-density cholesterol, LDL-C, low-density cholesterol, MDA, malondialdehyde, aNDF, neutral detergent fiber, SBO, soybean oil, SOD, superoxide dismutase, T-AOC, total antioxidant capacity, TC, total cholesterol, TG, total triglyceride, TP, total protein, UREA, serum urea nitrogen, Available energy content, Apparent total tract digestibility, Growing pigs, Chili meal, Growth performance

## Abstract

The objective of this study was to evaluate the digestible energy (DE), metabolizable energy (ME) content, apparent total tract digestibility (ATTD) of nutrients in chili meal (CM), and to determine the effects of CM on the performance of growing pigs. In Exp. 1, 12 barrows (Duroc x Landrace x Yorkshire) with an initial body weight (BW) of 50.9 ± 1.8 kg were allocated to one of two treatments, corn-soybean meal basal diet or diet containing 194.2 g/kg CM, which replaced corn and soybean meal in the basal diet. Pigs were placed in metabolism crates for a 7-d adaptation period followed by a 5-d total collection of feces and urine to detect DE, ME and ATTD of nutrients in CM. Exp. 2 was conducted for 4 wk. to evaluate the effect of CM on performance of growing pigs. 150 growing pigs (58.4 ± 1.2 kg BW) were allocated to 1 of 5 treatments. Treatment 1 was a corn-soybean meal basal diet met the DE requirement for growing pigs recommended by NRC (2012). Treatment 2 or 3 were diets containing 50 g/kg or 100 g/kg CM respectively. Treatment: 4 or 5 were based on treatment 2 or 3, while soybean oil (SBO) was added to improve the DE content to that in treatment 1. In Exp. 1, the DE and ME content of CM were 9.08 and 8.48 MJ/kg. The ATTD of dry matter (DM), gross energy (GE), organic matter (OM) and neutral detergent fiber (NDF) were 0.60, 0.54, 0.66 and 0.38, respectively. In Exp. 2, addition of CM linearly decreased (*P <* 0.05) average daily gain (ADG) and the ATTD of DM, GE and OM while ATTD of crude protein (CP) had a quadratic (*P <* 0.05) change. When SBO was supplemented in diets containing CM, greater values (*P <* 0.05) of ATTD of most nutrients were observed. With the dietary inclusion of CM, the albumin/globulin ratio in serum had a quadratic change (*P <* 0.05), and the level of low-density cholesterol linearly (*P <* 0.05) increased. In treatments with 50 g/kg CM, a significant reduction (*P <* 0.05) of total antioxidant capacity was found in diet formulated with SBO. In treatments with 100 g/kg CM, the level of total cholesterol was lower (*P <* 0.05) in the diet with SBO. In conclusion, CM had moderate energy density and nutrients digestibility in pig diets. 50 g/kg CM with SBO in diets could be fed to growing pigs with no significant negative effects.

## Highlights

• Chili meal is classified as a roughage due to its high dietary fiber content.
• Chili meal has moderate DE, ME and nutrients digestibility for growing pigs.
• Addition of chili meal have adverse effect on ADG and ATTD of nutrients.
• The combination of 50 g/kg chili meal and proper soybean has no significant negative effects for growing pigs.

## 1 Introduction

Chili, the capsicum fruit, is a kind of popular vegetables. It can be eaten freshly or processed to chili powder and chili jam etc. The chemical composition of chili has been reasonably well documented. Abundant nutrients in chili include vitamins (C, E), β-carotene and carotenoid pigments (Palevitch and Craker, 1996), which appear to be critically important in preventing chronic and age-related diseases (Minguez-Mosquera and Hornero-Mendez, 1994). In recent years, a growing number of chili was processed deeply to extract capsaicinoids, because they are important in the pharmaceutical industry for treatment of neurological disorders. Chili meal (CM), as the by-product of capsicum oleoresin extraction, is a potential source of feed material for abundant nutrients and increasing output.

In 2014, the world production of chili was 462955 ton and as the biggest producer, consumer and exporting country, the overall output in China was 32891 ton (FAO). When capsaicin is extracted form dried chili peel, more than 80% CM was left. However, being lack of research, most of the CM was ignored and discarded which leads to severe wasting of resources and environmental pollution. It is very important and necessary to develop CM as a new source of feed, because it can promote the utilization of CM and reduce the feeding cost. The research about CM in swine production seem empty in the world and vast research is needed to exploit potentialities of CM. Firstly, a digestion and metabolism experiment is wanted to provide an essential data about utilizability of CM in swine diet. Furthermore, Goncalves et al. (2012) reported that Brazilian red pepper meal (BRPM) contains tannins thus it needs to be evaluated through liver function and animal performance. When 78.9 g/kg CM was fed to broilers reared under high stocking density, greater growth performance and lower malondialdehyde (MDA) in serum were observed (Thiamhirunsopit et al., 2014). It suggested that CM can enhance antioxidant capacity for broilers. In order to study the effect of CM in swine production, a feeding experiment was conducted to evaluate the growth performance, nutrients digestibility, antioxidant index and conventional physiological and biochemical indexes in serum.

Therefore, the objective of this study was to evaluate the feeding value of CM for growing pigs and played a guidance role when it was applied in production.

## 2 Materials and methods

All procedures used in these experiments were approved by the Institutional Animal Care and Use Committee of China Agricultural University (Beijing, China). These experiments were conducted in the Metabolism Laboratory and Finishing Facility of the Fengning Pig Experimental Base (Hebei Province, China).

The processing methods of CM in China are similar and the main difference is the organic solvent when capsicum oleoresin was extracted. CM in present experiment was purchased from Chenguang Biotech Group Co., Ltd (Handan, China) and the processing flow was as follows: fresh chili was air dried, ground, and the husk of the chili was pelleted after seed-and-husk separation. Acetone was used with the pelleted chili husks to extract the capsaicin and the residue was dried. The dried residue is referred to as CM. The chemical composition of CM is shown in Table 1.

**Table 1;:** Chemical composition of chili meal (g/kg, dry matter basis).

Items	Chili meal
Dry matter	892.5
Crude protein	177.7
Crude fiber	246.8
Neutral detergent fiber	375.4
Acid detergent fiber	313.8
Ash	110.3
Calcium	3.92
Total phosphorus	3.70
Ether extract	4.03
Gross energy (MJ/kg)	18.91

### 2.1 Animals, diets and experimental designs

Exp. 1 was conducted to evaluate the digestible energy (DE), metabolizable energy (ME) content and apparent total tract digestibility (ATTD) of nutrients in CM. Twelve barrows (Duroc x Landrace x Yorkshire) with an initial average body weight (BW) of 50.9 ± 1.8 kg were housed individually in stainless steel metabolism crates (1.4 × 0.7 × 0.6 m^3^) and randomly allocated to one of two treatments. The basal diet contained 971.0 g/kg corn-soybean meal, and the treatment diet was formulated to contain 194.2 g/kg CM which replaced corn and soybean meal in the basal diet (Table 2). All the pigs were fed at 4% of their initial BW determined 1 day before the trial, and the daily feed allotment was divided into two equal portions which were fed at 08:30 and 15:30 h. All the pigs were allowed *ad libitum* access to water through a nipple waterer located at the front of the crate. The room temperature was maintained at 20 ± 1 °C and this experiment lasted for 12 days.

**Table 2:** Experimental diet composition and nutrient content in Exp. 1 (as-fed basis).

Item	Basal diet	Chili meal diet
Ingredients (g/kg)		
Corn	738.6	590.9
Soybean meal	232.4	185.9
Chili meal	0.00	194.2
Dicalcium phosphate	13.00	13.00
Limestone	8.00	8.00
Sodium chloride	3.00	3.00
Vitamin-mineral premix^a^	5.00	5.00
Chemical composition (g/kg)		
Dry matter	876.9	877.7
Crude protein	167.7	157.3
Ether extract	21.37	20.13
Neutral detergent fiber	142.6	172.8
Acid detergent fiber	55.31	89.84
Calcium	7.36	7.37
Total phosphorus	5.49	5.41
Gross energy (MJ/kg)	15.75	15.88
Organic matter	830.8	819.7

a Vitamin A, 5512 IU; vitamin D_3_, 2200 IU; vitamin E, 30 IU; vitamin K_3_, 2.2 mg; vitamin B_12_, 27.6 mg; riboflavin, 4 mg; pantothenic acid, 14 mg; niacin, 30 mg; choline chloride, 400 mg; folacin, 0.7 mg; thiamine, 1.5 mg; pyridoxine, 3 mg; biotin, 44 mg; Mn, 40 mg (MnO); Fe, 75 mg (FeSO_4_·H_2_O).

In Exp. 2, 150 crossbred barrows and gilts (Duroc x Landrace x Yorkshire) weighing 58.4 ± 1.2 kg BW were used in a 28-d experiment. Pigs were allocated to 1 of 5 treatments on the basis of weight and gender in a completely randomized design with 6 replicates (pens) per treatment and 5 pigs per pen. The pigs were housed in pens of 2.6 × 1.8 × 0.9 m^3^ with half of the floor cement and the other half woven mesh. All pigs had free access to water and feed throughout the 28-d experiment period. The temperature of the barn was set at 25 °C. Diets composition and nutrients concentration in Exp. 2 are presented in Table 3. Treatment 1 was corn-soybean meal basal diet which met the DE requirement for growing pigs recommended by NRC (2012). Treatment 2 or 3 were diets containing 50 g/kg or 100 g/kg CM respectively, which replaced corn and soybean meal in the basal diet. Treatment 4 or 5 were based on treatment 2 or 3, while soybean oil (SBO) was added to improve the DE content to that in treatment 1. Premix and amino acids were designed to meet the NRC (2012) requirements. DE of CM was from the result of Exp. 1 and DE of amino acids were from NRC (2012), while DE of corn and soybean meal were calculated in accordance with the prediction equations got in our lab: DE for corn [kcal/kg DM] = 1062.68 + 49.72EE[%]-24.89aNDF[%] + 0.54GE[MJ/kg DM] + 9.11Starch[%] (Li et al., 2014) and DE for soybean meal [MJ/kg DM] = 38.44-0.43CF[%]-0.98GE[MJ/kg DM] + 0.11ADF[%] (Li et al., 2016). Pigs were weighed at the beginning and the conclusion of the trial. The amount of feed offered to each pen was recorded, and at the end of the experiment (d 28), the amount of feed left in the feeder was weighted and used to calculate feed disappearance. The average daily gain (ADG), average daily feed intake (ADFI), feed: gain ratio (F: G) were calculated. As an exogenous indicator, acid-insoluble ash (AIA) in feed and feces were analyzed to calculate the digestibility coefficients of nutrients. Antioxidant indexes, physiological and biochemical indexes of serum were detected.

**Table 3:** Experimental diet composition and nutrient content in Exp. 2 (as-fed basis).

	Basal diet	50 g/kg Chili meal	100 g/kg Chili meal	50 g/kg Chili meal + Soybean oil	100 g/kg Chili meal + Soybean oil
Ingredients (g/kg)					
Corn	742.9	704.4	665.9	695.2	647.4
Soybean meal	222.0	210.5	199.0	207.7	193.5
Chili meal	0.00	50.0	100.0	50.0	100.0
Dicalcium phosphate	12.8	12.70	12.80	12.40	11.70
Limestone	6.70	6.40	5.80	6.40	6.50
Lysine (98%)	1.92	2.21	2.50	2.34	2.75
DL-methionine (98%)	0.22	0.29	0.36	0.32	0.42
Threonine (98%)	0.49	0.55	0.61	0.62	0.76
Soybean oil	0.00	0.00	0.00	12.00	24.00
Sodium chloride	3.00	3.00	3.00	3.00	3.00
Vitamin-mineral premix^a^	10.00	10.0	10.0	10.00	10.0
Digestible energy (kcal/kg)	3415	3350	3284	3413	3411
Analyzed chemical content (g/kg)					
Dry matter	871.0	872.6	872.7	872.9	875.5
Crude protein	156.8	151.8	153.7	159.7	156.9
Ether extract	31.4	29.24	26.62	39.07	48.91
Neutral detergent fiber	155.2	157.0	164.2	158.4	163.0
Acid detergent fiber	52.03	50.32	65.67	63.59	64.2
Calcium	5.18	5.56	5.22	5.24	5.44
Total phosphorus	4.90	4.95	5.31	4.88	4.82
Gross energy (MJ/kg)	160.6	159.9	160.4	162.9	166.3
Organic matter	829.2	827.5	824.6	828.3	828.7

a Vitamin A, 5512 IU; vitamin D_3_, 2200 IU; vitamin E, 30 IU; vitamin K_3_, 2.2 mg; vitamin B_12_, 27.6 mg; riboflavin, 4 mg; pantothenic acid, 14 mg; niacin, 30 mg; choline chloride, 400 mg; folacin, 0.7 mg; thiamine, 1.5 mg; pyridoxine, 3 mg; biotin, 44 mg; Mn, 40 mg (MnO); Fe, 75 mg (FeSO_4_·H_2_O).

### 2.2 Sample collection

In Exp. 1, after a 7-d-adaptation period, a 5-d-total collection of feces and urine was conducted. Feed refusals and feed spillage were collected, dried and weighed, and feed intake was calculated. Feces were collected immediately as they appeared in the metabolism crates, placed in plastic bags and stored at −20 °C. Urine was collected in a bucket placed under the metabolism crates. The bucket contained 10 mL of 6 N HCl for every 1000 mL of urine to avoid the loss of nitrogen. Each day, the total urine volume was measured, then a 10% aliquot of urine was filtered through gauze and pooled together per pig, and 50 mL of the mixed urine sample was transferred into a screw-capped tube and immediately stored at −20 °C. At the end of the collection period, the sampled feces and urine were pooled for each pig and subsamples were collected for chemical analysis. The subsamples of feces were dried for 72 h at 65 °C and ground through a 1-mm screen.

In Exp. 2, Feces was sampled on d 27–28 for all the pigs and mixed by each replicate, then dried by the same method as Exp. 1. Blood samples (1 pig/replicate) were collected from pigs after a 12 h fast at 8:00 am via the superior vena cava into vacuum blood collection tubes on d 29. The blood samples were centrifuged at 3500 × *g* for 15 min at 4 °C (Ciji 800 Model Centrifuge; Surgical Instrument Factory, Changzhou, China) and the serum was stored at −20 °C for analysis.

### 2.3 Chemical analysis and calculation

All chemical analyses were conducted in duplicate. Samples of CM, diets and feces in both two experiments were analyzed for dry matter (DM; AOAC, 2007 method 930.15), crude protein (CP; AOAC, 2007 method 984.13), ether extract (EE; AOAC, 2000 method 920.39), ash (AOAC, 2007 method 942.05), calcium (Ca; AOAC, 2007 method 927.02), total phosphorus (P; AOAC, 2007 method 984.27) and crude fiber (CF; AOAC, 2006 method 978.10). Organic matter (OM) was computed as 100 minus the content of ash and water. Neutral detergent fiber (aNDF) and acid detergent fiber (ADF) were determined using fiber filter bags and fiber analyzer equipment (Fiber Analyzer, Ankom Technology, Macedon, NY) following an adaptation of the procedure described by Van Soest et al. (1991). The concentration of aNDF was analyzed using heat stable α-amylase and sodium sulfite without correction for insoluble ash (inclusive of residual ash). The ADF fraction was analyzed in a separate sample (inclusive of residual ash). Samples of CM, diets, feces and urine were analyzed for gross energy (GE) with an Isoperibol Oxygen Bomb Calorimeter (Parr 6400 Calorimeter, Moline, IL). The diets and feces in Exp. 2 were analyzed for AIA by the methods described by McCarthy et al. (1974).

The levels of malondialdehyde (MDA), superoxide dismutase (SOD) and total antioxidant capacity (T-AOC) in serum were determined by commercial kits (Jiancheng Biochemical Reagent Co., Nanjing, China) according to the manufacturer’s instructions. The concentration of albumin (ALB), globulin (GLB), total protein (TP), low-density cholesterol (LDL-C), high-density cholesterol (HDL-C), total cholesterol (TC), calcium (Ca), total phosphorus (P), glutamic-pyruvic transaminase (ALT), glutamic oxalacetic transaminase (AST), creatinine (CREA), alkaline phosphatase (ALP), serum urea nitrogen (UREA), glucose (GLU) and total triglyceride (TG) in serum were measured by corresponding commercial kits (BioSino Bio-technology and Science Inc., Beijing, China) using an automatic biochemical analyzer (Hitachi 7160, Hitachi High-Technologies Corporation, Japan).

In Exp. 1, DE and ME content of the CM were calculated using the difference method (Adeola, 2001). The ATTD of nutrients in diets was determined by following equation (Kong and Adeola, 2014):

D _ti_ = {D _td_ − [D _bd_ × (1–P _ti_)]}/P _ti_

In this equation, D _bd_, D _td_ and D _ti_ are the digestibility (%) of the component in the basal diet, test diet, and test ingredient (CM), respectively, and P _ti_ are the proportional contribution of the component by the test ingredient to the test diet, respectively.

In Exp. 2, the ATTD of nutrients were calculated as follows (Kong and Adeola, 2014):

Digestibility (%) = 100-[(CI _input_ × CC _output_)/(CI _output_ × CC _input_) × 100]

In this equation, CI _input_ and CI _output_ are the concentration of index compound (AIA) in feed and feces, respectively; CC _input_ and CC _output_ are the concentration of component in feed and feces, respectively.

In these two experiments, digestibility coefficients were then determined by dividing grams of component digested by the grams of component consumed.

### 2.4 Statistical analysis

Data were checked for normality and outliers were detected using the UNIVARIATE procedure of SAS (SAS Institute, Cary, NC). No outliers were identified. In Exp. 1: Data were analyzed using the PROC GLM procedure of SAS (SAS Institute). Pig was treated as the experimental unit. One-way ANOVA was used for data analysis. Treatment means were calculated using the LSMEANS statement and statistical differences among the treatments were separated by SNK test. In Exp. 2: Pen was treated as the experimental unit and two methods were used to data analysis. Orthogonal contrast was used to determine the effect of 50 g/kg CM (-SBO) vs. 50 g/kg CM (+SBO), 100 g/kg CM (-SBO) vs. 100 g/kg CM (+SBO) and basal diet vs. 50 g/kg CM (+SBO) vs. 100 g/kg CM (+SBO). Polynomial contrasts were conducted to determine linear and quadratic effects of basal diet vs. 50 g/kg CM (-SBO) vs. 100 g/kg CM (-SBO). Significant differences were declared at P < 0.05. Differences at 0.05 ≤ P < 0.10 were considered as a trend toward significance.

## 3 Results

### 3.1 Experiment 1. Nutrients concentration, digestibility, digestible and metabolizable energy content in chili meal

The GE in CM was 18.91 MJ/kg (DM basis; Table 1). The concentration of CP, ash, and crude fiber (CF) were 177.7, 110.3 and 246.8 g/kg respectively. There was no significant difference in the GE intake between the basal diet and CM diet (Table 4). The CM diet fed group had greater (*P <* 0.01) dry feces output, GE in dry feces, and fecal GE output than the basal diet group. As a consequence, values for DE, ME, and ME/DE, DE/GE and ME/GE ratio were less (*P <* 0.05) in the CM diet, and pigs fed the CM diet had lower (*P <* 0.01) ATTD of DM, CP, aNDF, ADF, Ca, GE, and OM compared with pigs fed the basal diet (Table 5). However, there was a trend that CM diet had higher (0.05 < *P <* 0.10) ATTD of EE than basal diet for growing pigs. The DE and ME content of CM were 9.08 and 8.48 MJ/kg (as-fed basis), and the ME/DE ratio was 0.93. The ATTD of DM, aNDF, ADF, GE, and OM were 0.60, 0.38, 0.33, 0.54 and 0.66, respectively.

**Table 4:** Energy utilization^a^ of experimental diets when fed to growing pigs Exp. 1.

Item	Basal diet	Chili meal diet	SEM	P-value
Diet				
Total feed intake (kg)	10.09	9.69	0.31	0.419
Gross energy intake (MJ)	158.83	153.94	4.95	0.522
Dry feces output (kg)	0.9	1.39	0.08	0.002
Gross energy in dry feces (MJ/kg)	17.53	19.40	0.10	<0.001
Fecal Gross energy output (MJ)	15.95	27.00	1.37	<0.001
Urine output (kg)	26.90	31.10	8.24	0.739
Gross energy in urine (MJ/kg)	0.14	0.13	0.03	0.926
Urinary gross energy output (MJ)	2.70	3.20	0.22	0.160

a The observation of each treatment is six.

**Table 5:** Energy values and digestibility of various chemical constituents^a^ of experimental diets and chili meal when fed to growing pigs in Exp. 1 (as-fed basis).

Item	Basal diet	Chili meal diet	SEM	*P*-value	Chili meal
Digestible energy(MJ/kg)	14.18	13.10	0.08	<0.001	9.08
Metabolizable energy(MJ/kg)	13.91	12.77	0.09	<0.001	8.48
Metabolizable energy/Digestible energy	0.98	0.97	<0.01	0.017	0.93
Digestible energy/Gross energy	0.90	0.83	<0.01	<0.001	0.54
Metabolizable energy/Gross energy	0.88	0.80	<0.01	<0.001	0.50
Dry matter	0.90	0.84	<0.01	<0.001	0.60
Crude protein	0.87	0.78	0.01	0.002	0.31
Ether extract	0.56	0.61	0.01	0.078	0.86
Neutral detergent fiber	0.73	0.61	0.02	0.002	0.38
Acid detergent fiber	0.76	0.55	0.02	<0.001	0.33
Total phosphorus	0.59.	0.57	0.02	0.583	0.49
Gross energy	0.90	0.83	<0.01	<0.001	0.54
Organic matter	0.92	0.85	<0.01	<0.001	0.66

a The observation of each treatment is six.

### 3.2 Experiment 2. Effects of chili meal on growth performance and nutrients digestibility for growing pigs

No significant differences were observed in ADFI and F:G among treatments (Table 6). With the inclusion of CM, the ADG and ATTD of DM, GE, and OM linearly (*P <* 0.05) decreased with a trend for quadratic decrease (0.05 <* P <* 0.10; 0.53 vs. 0.33 vs. 0.38) for ADF, and the ATTD of CP changed quadratically (*P <* 0.05; 0.87 vs. 0.85 vs. 0.86; Table 7). When SBO was fed with 50 g/kg CM, there was a trend that ME intake and ADG were improved (0.05 < *P <* 0.10), and the ATTD of DM, CP, EE, aNDF, ADF, GE and OM were greater (*P <* 0.05) in the diet formulated with SBO. In 100 g/kg CM treatments, the ATTD of DM, EE, GE, and OM were greater (*P <* 0.05) when SBO was added. Compared with control treatment, the ATTD of EE were greater (*P <* 0.01) in two SBO treatments, but the ATTD of CP in 100 g/kg CM treatment with SBO was lower than control or 50/kg CM treatments with SBO (*P <* 0.01).

**Table 6:** Effect of chili meal on growth performance^a^ for growing pigs in Exp. 2.

	Basal	50 g/kg CM^b^		100 g/kg CM		SEM	*P*-value	*P*-value (CM)		*P*-value (+/−SBO)		*P*-value (DE)^h^
		-SBO^c^	+SBO	-SBO	+SBO			Linear^d^	Quadratic^e^	50 g/kg CM^f^	100 g/kg CM^g^	
Average daily gain (kg/d)	1.24	1.07	1.23	1.07	1.16	0.05	0.031	0.007	0.082	0.055	0.106	0.573
Average daily feed intake (kg/d)	2.46	2.26	2.51	2.33	2.41	0.11	0.524	0.439	0.361	0.145	0.544	0.841
Feed: gain	1.99	2.13	2.06	2.19	2.08	0.11	0.778	0.273	0.361	0.668	0.479	0.813
DE intake (kcal/d)	8400	7564	8557	7643	8230	374.55	0.249	0.196	0.360	0.092	0.222	0.837

a The observation of each treatment is six.

b CM = chili meal.

c SBO = soybean oil.

d Linear = Control vs. 50 g/kg CM (-SB0) vs. 100 g/kg CM (-SBO).

e Quadratic = Control vs. 50 g/kg CM (-SBO) vs. 100 g/kg CM (-SBO).

f 50 g/kg CM = 5 g/kg CM (-SBO) vs. 50 g/kg CM (+SBO).

g 100 g/kg CM = 100 g/kg CM (-SBO) vs. 100 g/kg CM (+SBO).

h P-value (DE) = Control vs. 50 g/kg CM (+SBO) vs. 100 g/kg CM (+SBO).

**Table 7:** Effect of chili meal on digestibility of nutrients^a^ when fed to growing pigs in Exp. 2.

	Basal	50 g/kg CM^b^		100 g/kg CM		SEM	*P*-value	*P*-value (CM)		*P*-value (+/−SBO)		*P*-value (DE)^h^
		-SBO^c^	+SBO	-SBO	+SBO			Linear^d^	Quadratic^e^	50 g/kg CM^f^	100 g/kg CM^g^	
DM^i^	0.82	0.80	0.83	0.79	0.83	0.07	0.003	0.039	0.670	0.009	<0.001	0.517
CP^j^	0.87^a^	0.85	0.87^a^	0.86	0.85^b^	0.03	<0.001	0.012	0.018	<0.001	0.826	0.002
EE^k^	0.30^b^	0.33	0.55^a^	0.25	0.63^a^	0.29	<0.001	0.291	0.232	<0.001	<0.001	<0.001
aNDF^l^	0.48	0.46	0.56	0.49	0.57	0.30	0.100	0.751	0.621	0.004	0.157	0.114
ADF^m^	0.53	0.33	0.53	0.38	0.48	0.39	0.004	0.054	0.060	<0.001	0.120	0.582
GE^n^	0.81	0.79	0.82	0.77	0.82	0.09	0.002	0.007	0.800	0.017	<0.001	0.886
OM^o^	0.85	0.83	0.85	0.81	0.85	0.07	0.004	0.009	0.623	0.019	0.002	0.965

a The observation of each treatment is six.

b CM = chili meal.

c SBO = soybean oil.

d Linear = Control vs. 50 g/kg CM (-SB0) vs. 100 g/kg CM (-SBO).

e Quadratic = Control vs. 50 g/kg CM (-SBO) vs. 100 g/kg CM (-SBO).

f 50 g/kg CM = 50 g/kg CM (-SBO) vs. 50 g/kg CM (+SBO).

g 100 g/kg CM = 100 g/kg CM (-SBO) vs. 100 g/kg CM (+SBO).

h P-value (DE) = Control vs. 50 g/kg CM (+SBO) vs. 100 g/kg CM (+SBO).

i DM = dry matter.

j CP = crude protein.

k EE = ether extract.

l aNDF = neutral detergent fiber.

m ADF = acid detergent fiber.

n GE = gross energy.

o OM = organic matter.

### 3.3 Experiment 2. Effects of chili meal on antioxidant indexes, physiological and biochemical indexes of serum for growing pigs

Neither CM nor SBO affected the levels of MDA and SOD in serum (*P >* 0.10; Table 8). With increasing levels of CM in diets, the value for ALB/GLB (A/G) changed quadratically (*P <* 0.05; 0.86 vs. 0.66 vs. 0.75), and the level of LDL-C increased linearly (P < 0.05). There was also a trend that CM linearly increased (0.05 <* P <* 0.10) the level of TC and changed the value of AST/ALT ratio quadratically (0.05 <* P <* 0.10; 0.68 vs. 1.09 vs. 0.84). When SBO was fed with 50 g/kg CM, a reduction (*P <* 0.05) of T-AOC and a trend of reduction (0.05 < *P <* 0.10) for CREA were found. In 100 g/kg CM treatments, the TC was lower (*P <* 0.05) in pigs fed with SBO compared to those without SBO, and there was a trend that SBO decreased (0.05 <* P < *0.10) the LDL-C and Ca levels. Pigs fed with two SBO diets had a trend (0.05 <* P < *0.10) to increase AST/ALT ratio than control diet.

**Table 8:** Effect of chili meal on serum antioxidant indicators, physiological and biochemical indicators^a^ in Exp. 2.

	Basal	50 g/kg CM^b^		100 g/kg CM		SEM	*P*-value	*P*-value (CM)		*P*-value (+/−SBO)		*P*-value (DE)^h^
		-SBO^c^	+SBO	-SBO	+SBO			Linear^d^	Quadratic^e^	50 g/kg CM^f^	100 g/kg CM^g^	
Antioxidant indicators												
MDA^i^(nmol/ml)	2.47	1.90	2.35	2.29	2.93	0.35	0.365	0.657	0.178	0.267	0.340	0.535
SOD^j^ (NU/ml)	129.89	137.56	120.83	119.48	124.92	6.83	0.349	0.285	0.134	0.179	0.561	0.605
T-AOC^k^ (NU/ml)	2.25	2.51	1.69	2.27	1.77	0.25	0.127	0.961	0.497	0.022	0.197	0.194
Physiological and biochemical indicators												
ALT^l^(U/L)	41.67	41.33	44.00	48.83	44.67	4.06	0.701	0.295	0.504	0.631	0.531	0.781
AST^m^ (U/L)	28.50	41.67	42.50	42.33	29.83	5.93	0.247	0.132	0.418	0.925	0.206	0.136
AST/ALT	0.68	1.09	0.94	0.84	0.67	0.12	0.123	0.420	0.080	0.498	0.320	0.070
ALB^n^ (g/L)	38.32	36.15	36.48	39.60	36.00	1.64	0.461	0.616	0.215	0.884	0.207	0.451
GLB^o^ (g/L)	46.23	54.95	48.57	53.70	47.67	3.26	0.261	0.158	0.270	0.126	0.241	0.873
A/G^p^	0.86	0.66	0.76	0.75	0.77	0.05	0.117	0.145	0.047	0.151	0.732	0.361
TP^q^ (g/L)	84.55	91.10	85.05	93.30	83.67	4.13	0.371	0.187	0.697	0.241	0.199	0.962
LDL-C^r^(mmol/L)	1.58	1.66	1.68	2.05	1.55	0.13	0.080	0.043	0.412	0.873	0.071	0.548
HDL-C^s^(mmol/L)	0.56	0.58	0.61	0.64	0.51	0.06	0.565	0.420	0.751	0.708	0.168	0.284
TC^t^(mmol/L)	2.66	2.83	2.87	3.27	2.51	0.19	0.093	0.068	0.613	0.869	0.049	0.176
Ca^u^ (mmol/L)	2.39	2.37	2.43	2.66	2.35	0.10	0.180	0.118	0.310	0.603	0.091	0.629
P^v^ (mmol/L)	3.17	2.99	3.03	3.17	2.94	0.17	0.809	0.996	0.488	0.881	0.370	0.360
TG^w^(mmol/L)	0.35	0.65	0.57	0.60	0.62	0.12	0.405	0.150	0.240	0.726	0.903	0.157
CREA^x^ (umol/L)	116.12	124.02	106.22	136.65	121.67	8.22	0.152	0.133	0.835	0.062	0.294	0.365
ALP^y^ (U/L)	129.50	91.00	133.50	116.83	77.50	23.71	0.394	0.722	0.305	0.228	0.125	0.285
UREA^z^ (mmol/L)	4.60	4.25	5.50	4.65	4.20	0.65	0.632	0.959	0.656	0.154	0.681	0.303
GLU^A^ (mmol/L)	5.75	5.30	5.96	6.08	5.22	0.40	0.462	0.598	0.272	0.263	0.211	0.270

a The observation of each treatment is six.

b CM = chili meal.

c SBO = soybean oil.

d Linear = Control vs. 50 g/kg CM (-SB0) vs. 100 g/kg CM (-SBO).

e Quadratic = Control vs. 50 g/kg CM (-SBO) vs. 100 g/kg CM (-SBO).

f 50 g/kg CM = 50 g/kg CM (-SBO) vs. 50 g/kg CM (+SBO).

g 100 g/kg CM = 100 g/kg CM (-SBO) vs. 100 g/kg CM (+SBO).

h P-value (DE) = Control vs. 50 g/kg CM (+SBO) vs. 100 g/kg CM (+SBO).

i MDA = malondialdehyde.

j SOD = superoxide dismutase.

k T-AOC = total antioxidant capacity.

l ATL = glutamic-pyruvic transaminase.

m AST = glutamic oxalacetic transaminase.

n ALB = albumin.

o GLB = globulin.

p A/G = albumin/globulin ratio.

q TP = total protein.

r LDL-C = low-density cholesterol.

s HDL-C = high-density cholesterol.

t TC = total cholesterol.

u Ca = calcium.

v P = total phosphorus.

w TG = total triglyceride.

x CREA = creatinine.

y ALP = alkaline phosphatase.

z UREA = serum urea nitrogen.

A GLU = glucose.

## 4 Discussion

### 4.1 Nutrients content, DE, ME, and nutrients digestibility of chili meal

The concentration of CP and ash in CM were 177.7 and 110.3 g/kg in this experiment, which were greater than the values (150.0 and 80.3 g/kg, respectively) reported by Thiamhirunsopit et al. (2014), but the concentrations of CF, Ca, P and EE (246.8, 3.92, 3.70 and 4.03 g/kg, respectively) in this study were lower than those in their report (253.0, 20.3, 6.9, 115 g/kg, respectively). This discrepancy might result from factors, such as different varieties and environments under which the chili was grown, stored and processed into CM. In their study, CM was the by-product of ethanol extraction of whole dried chili which included the chili seeds. However, the CM used in our experiment was the residue of chili husk after acetone extraction. This might imply that chili husk had greater CP but lesser Ca, P and oil content than chili seed.

Chili meal is classified as a roughage due to its high dietary fiber content. Total dietary fiber primarily consists of non-starch polysaccharides, which are well known to encapsulate nutrients, and monogastric animals do not have proper enzymatic ability to digest such complex structures (Bedford and Schulze, 1998). Therefore, an increasing fiber content is negatively correlated with digestibility and the energy content of feedstuffs (Noblet and Le Goff, 2001). In the present study, the inclusion of CM with high fiber content in diets increased the concentration of aNDF and ADF compared with the corn-soybean meal basal diet, which resulted to the increase of fecal output. This is consistent with previous reports (Wilfart et al., 2007) and related to the water holding capacity of soluble dietary fiber and increase in fecal bulk of insoluble dietary fiber (Serena et al., 2008). Therefore, higher fecal gross energy out resulted in lower DE and ME content and nutrients digestibility.

### 4.2 Effect of chili meal on growth performance and nutrients digestibility for growing pigs

In this research, the DE of diets in treatments 2 and 3 formulated with 50 g/kg and 100 g/kg CM were lower than the energy requirements due to lower energy content in CM. No significant difference in ADFI was observed among the treatments and lower DE intake was observed for pigs fed the CM diets. In growing pigs, it has been previously reported that 1% increase in dietary NDF reduced the energy digestibility by 0.9% (Le Goff and Noblet, 2001), because the dietary increase of NDF may stimulate bowel movement and reduce the transit time of digesta (Bindelle et al., 2008, Bastianelli et al., 1996). Consistent with those reports, lesser ATTD of DM, CP, GE and OM was evident in pigs fed CM diets. Therefore, the ADG of pigs linearly decreased with the dietary inclusion of CM. The concentrations of CP, aNDF and ADF in alfalfa meal (162.5, 420.0 and 321.5 g/kg, respectively) are similar to those of CM (NRC, 2012). In research with alfalfa meal, Chen et al. (2014) reported that ADG and the ATTD of DM, OM, CP, NDF, ADF and GE reduced linearly as the level of alfalfa meal in the diet increased (50, 100 and 200 g/kg).

### 4.3 Effect of soybean oil on growth performance and nutrients digestibility for growing pigs

Increased dietary fiber level is associated with a reduced available energy content in feed, in most practical conditions, fiber-rich ingredients are combined with high energy ingredients such as animal fat or vegetable oil in order to maintain the dietary energy level (Bakker, 1996, Noblet and Shi, 1994). Therefore, in the present experiment, SBO were added in treatments 4 and 5 to improve DE content to required levels. Addition of soybean oil contributed to a reduced rate of gastric emptying (Gentilcore et al., 2006), and increased the retention time of digesta in intestinal tract (Cervantes-Pahm and Stein, 2008), which increased the fermentability of fiber due to a longer exposure of substrates to the intestinal microbiota (Wilfart et al., 2007, Morel et al., 2006). Therefore, addition of soybean oil can increase digestibility of energy. Our research results showed that addition of SBO in CM diets improved the ATTD of most nutrients compared to diets without SBO. Moreover, compared with the control treatment, the ATTD of EE were greater in the two SBO treatments. The increased ATTD of EE in diets by addition of SBO is a consequence of a higher digestibility of SBO compared to corn and soybean meal, because intact fat from corn and soybean meal is encased with fat cell membranes and thus is more resistant to the formation of emulsions and enzymatic digestion than extracted fat from SBO (Kil et al., 2010). Another reason for that is the addition of SBO increased the EE content in diets and apparently digested fat was linearly related to dietary fat intake (Kil et al., 2010). Consistent with results from this research, other reports have shown that, with the dietary inclusion of SBO, the ATTD of DM, GE, AEE and NDF increased (Gutierrez et al., 2016, Du et al., 2009). Dietary inclusion of SBO with 50 g/kg CM tended to increase the ADG of growing pigs, which was consistent with the DE intake and the improvement of nutrients digestibility induced by SBO. Pettey et al. (2002) also reported that, when feeding a corn-dehulled soybean meal basal diet to finishing pigs, the addition of 20 g/kg SBO tended to improve F: G compared with the control. Conversely, Kil et al. (2013) indicated that addition of SBO had no effect on growth performance but increased ATTD of acid hydrolyzed ether extract and GE.

### 4.4 Effect of chili meal and soybean oil on serum antioxidant indicators, physiological and biochemical indicators of growing pigs

Thiamhirunsopit et al. (2014) found that CM in diets reduced the MDA level to the normal levels when broilers were under high stocking density. In their study, CM contained 0.43 g/kg capsaicin, an important alkaloid due to its neurological benefit, and potential lipid peroxidation properties (Kentaro et al., 2002, Conforti et al., 2007). In present study, with the dietary inclusion of CM, no significant difference was observed in MDA, SOD or T-AOC. It is possible that acetone extraction was sufficiently efficient so that there was little remaining capsaicin in CM in the current study. However, when 50 g/kg CM were added to the diets, significant reduction of T-AOC was found in diet formulated with SBO, which indicated that the inclusion of SBO might reduce antioxidant capacity. Total antioxidant capacity is considered to be the integrated action of all the antioxidants present in plasma and body fluids, thus providing an insight into a delicate balance in vivo between oxidants and antioxidants (Ghiselli et al., 2000). This is consistent with a report that high fat significantly elevated MDA levels and lowered T-AOC levels in the serum of rabbits (Sun et al., 2014).

Changes in serum biochemical indicators suggest the change in tissue cell permeability and body's metabolic function With the dietary inclusion of CM, no significant difference was observed in the serum concentrations of AST or ALT. The levels of the enzymes ALT and AST are tools in the diagnosis of liver function. Similar results was observed that the serum concentrations of AST and ALT in broiler chickens fed diet with 12 g/kg of Brazilian red pepper meal did not differ from the negative control.

Concentrations of TC, UREA and TP reflect the health and nutritional status of pigs (Etim et al., 2014). UREA concentration indicates protein catabolism. Increased TP concentration suggests that more protein is available for utilization and the decrease in UREA suggests sufficient protein consumption (Hlatini and Chimonyo, 2016). With the inclusion of CM, the level of A/G had a quadratic change, and the lowest numerical number was observed in the diet containing 50 g/kg CM. The decline of A/G ratio indicated that more globulin was synthesized to elevate the immune status of the pigs. With the inclusion of CM, the level of LDL-C increased linearly but the addition of SBO decreased the level of TC when 100 g/kg CM was used. Excessive cholesterol can contribute to arthrosclerosis and lead to coronary problems in humans. In pigs, the normal blood cholesterol range is 81–134 mg/dl (Radostits et al., 2000) and all the TC contents of serum in present study were lower than it. The decreased serum TC may be associated with a decline in lipid mobilization (Prvulovic et al., 2007).

## 5 Conclusion

The present study demonstrated that inclusion of chili meal in swine diets, as a novel fibrous feedstuff, results in moderate DE, ME and nutrients digestibility for growing pigs. The inclusion of chili meal decreases the average daily gain and digestibility of most nutrients. Supplementation of CM diets with soybean oil increases the average daily gain and digestibility of nutrients. The combination of 50 g/kg chili meal and proper soybean has no significant negative effects for growing pigs.
